# Memoranda of an Obstetrical Case

**Published:** 1869-12

**Authors:** J. W. Brooks

**Affiliations:** Chicago


### Article X.—*Memoranda of an Obstetrical Case.* By J. W. Brooks, M.D., Chicago.

Perhaps the following memoranda of an obstetrical case may not be uninteresting to the medical reader:

Was requested to visit Mrs. ——, of —— avenue, at one o'clock, A.M., October 24, 1869. Found slight labor pains in progress, at the full period of second pregnancy. Tactile examination discovered the head low in the pelvis, and no dilatation of the os uteri. Both lady and nurse assured me that no liquor amnii had passed off. Matters remained in *statu quo* until six and a half, A.M., when the pains abruptly quickened, and the os began to dilate. An examination now disclosed a mento posterior presentation, the anterior fontanelle being under the arch of the pubis. (The case is well illustrated in Smellie's 26th anatomical plate.) The frontal bones lapped at each recurring pain, and it being evident that the posterior portion of the cranium was above the arch of the pubis, and no progress being made in the labor, therefore, in the absence of a uterine contraction, I made an effort to change the presenting position, as follows: With two fingers of the right hand introduced, a trial was made to push the child back in order to change the direction and position of the head. The third was successful, and as the whole child receded, there was a simultaneous receding of the chin and a corresponding advance of the occiput, with partial rotation of the head, which brought it into the first natural position of Rigby, it being evidently the result of the effort to change the presentation. From this time the labor progressed naturally, and in one hour she was delivered of a fine, healthy girl, which occurred at eight fifteen, A.M. The secundines were delivered in twenty minutes. During the delivery of them something was found attached to the outside of the membranes, and also to the uterus, which was separated from the latter with the finger, and left on the former. A little, brisk hæmorrhage followed the detachment, which soon ceased. A critical examination of this body proved it to be a small liver. The uterus contracted firmly, but seemed larger than normal. I waited about two hours, and was on the point of leaving, when very moderate hæmorrhage commenced, and with each after-pain increased materially. Neither the application of cold, or the use of ergot, checked it. It increasing with great rapidity, I took one ounce of strong solution of perchloride of iron, and four ounces of tepid water, and filled a small gutta-percha syringe with the mixture, and fitted it to a male gum catheter, size twelve, with single eye, and prepared to introduce the catheter into the uterus to inject the fluid therein. Within the os a firm, resisting body was encountered. On opening the os the hæmorrhage was fearful. I proceeded to push the index finger onward, carrying the catheter with it. When the obstruction gave way the catheter was carried in and the whole fluid injected, which temporarily allayed the hæmorrhage. Finding something attached to the posterior part of the uterus that could be separated, proceeded at once to detach and remove it, which was successfully accomplished, but the hæmorrhage returned violently, and another injection of like kind, strength and quantity, was used, which checked the flooding. The offending cause proved to be another set of membranes, attached by a large surface; and between them and the walls of the uterus were evidently the remains of a blighted ovum. The head, heart and kidneys could be made out, and a good deal of shapeless muscular tissue, together with some points of ossification in this tissue. The placenta, etc., can still be seen. From this time no untoward event occurred, and Mrs. —— has made a good recovery.

When anxiety was at its height, it was announced by one who knew, that a certain Homœopathic professor had administered a little powder to remove what was believed to be a recent conception, at the same time saying that if it did not remove the thing it would do no harm. The remark was also made that she was somewhat unwell, for a day or two; that is, had some show, but went on with her pregnancy.

Query: Did the little powder have the effect to partially separate and blight one ovum, the conception having been twins?

